# Osteonecrosis of Bilateral Distal Femurs in a Pregnant Patient Following Antenatal Betamethasone

**DOI:** 10.7759/cureus.22735

**Published:** 2022-03-01

**Authors:** Rafal S Ali, Hussein Al-Sudani, Irene J Tan

**Affiliations:** 1 Internal Medicine, Einstein Medical Center Montgomery, East Norriton, USA; 2 Rheumatology, Einstein Medical Center Philadelphia, Philadelphia, USA

**Keywords:** steroid induced osteonecrosis, distal femur, pregnancy, steroid, avascular osteonecrosis

## Abstract

Corticosteroid therapy is a known risk factor for osteonecrosis, more commonly with chronic use and high cumulative dose. Osteonecrosis (avascular necrosis) has been described in pregnancy involving primarily the femoral head. To our knowledge, only rare cases of femoral meta diaphysis or knee osteonecrosis in pregnancy have been documented in the literature.

We report a 28-year-old woman with sickle cell trait and beta-thalassemia trait who developed severe bilateral knee pain shortly after corticosteroid therapy. She was 34-weeks pregnant when she presented with the signs of preterm labor and was found to have oligohydramnios and preeclampsia. She was given two intramuscular injections of betamethasone 12 mg one day apart to enhance the fetal lung maturity. Within hours of the second injection, she developed acute and severe bilateral knee pain affecting her mobility and ambulation. Bilateral knee x-rays were unremarkable. Given the severity and persistence of her pain, magnetic resonance imaging (MRI) of bilateral lower extremities was done few days later and showed signs of early osteonecrosis involving bilateral distal femoral meta diaphysis and right lateral femoral condyle.

Other than the steroid therapy she had received, no additional extrinsic risk factors for osteonecrosis were identified. Potential intrinsic risk factors were thought to include her combined sickle-beta-thalassemia traits and pregnancy. She was diagnosed with steroid-induced osteonecrosis, given the temporal relationship. Her presentation was unique, because osteonecrosis affected unreported sites during pregnancy, and it started shortly after a brief course of antenatal steroid. She was treated conservatively with analgesics, and outpatient orthopedic follow-up was recommended. She was advised to avoid prolonged weight-bearing and strenuous activities. On a follow-up appointment two months later, she was still complaining of bilateral knee pain with ambulation though it was less severe. She did not return for follow-up thereafter.

We suggest the possibility of osteonecrosis in pregnancy involving uncommon sites, such as distal femur and femoral condyle in this case, following one or two doses of systemic steroid. Obstetricians need to consider osteonecrosis when evaluating an unexplained musculoskeletal pain after betamethasone that is used for preterm labors. More studies, including reporting more cases with unusual presentation and prospective studies following pregnant patients receiving steroid therapy, are needed to better understand the causes, associations, management, and clinical course of osteonecrosis in pregnancy.

## Introduction

Osteonecrosis, also known as avascular necrosis, ischemic necrosis, and aseptic necrosis of bone, is a disabling condition involving bone cell death, followed by resorption that can be complicated by subchondral fracture, the possible collapse of an articular surface, and loss of joint function [1-3]. In the United States, there is an estimated incidence of 10,000 to 20,000 cases per year [4]. Osteonecrosis can be primary with no identifiable cause or secondary to traumatic and non-traumatic factors, with glucocorticoids and excessive alcohol intake comprising most of the non-traumatic osteonecrosis cases. Other risk factors include bisphosphonates, cigarette smoking, systemic lupus erythematosus (SLE), sickle cell disease, Gaucher disease, human immunodeficiency virus (HIV) infection, and radiation therapy.

Several mechanisms have been proposed for the pathogenesis of glucocorticoid-associated osteonecrosis [2,5]. One mechanism considered the possibility of micro-emboli involving the bone vascular supply [6]. Another proposed mechanism is increased pressure inside the bone secondary to increased bone marrow adipocyte size and number causing venous outflow obstruction [7]. An additional theory suggests that steroids can decrease the venous blood flow by changing venous endothelium that causes increased intraosseous pressure and death of bone cells [8].

Osteonecrosis, secondary to corticosteroids, most commonly affects the femoral head; other sites can also be involved such as bones around the knee joint, wrist joint, and ankle joint. Combined genetic and environmental factors can also play a role [9]. Prior reported osteonecrosis cases related to inhaled or topical use of steroids were confounded by the previous administration of systemic steroid therapy [10]. Low but statistically significant increased incidence of osteonecrosis has been reported in individuals who had short-term, low-dose oral steroid administration [11].

## Case presentation

A 28-year-old African American female, who is 34-weeks pregnant, presented to the obstetrical unit with increased vaginal discharge and intermittent contractions. She was found to have 4-cm cervical dilatation. Fetal ultrasound showed oligohydramnios, and her blood pressure was elevated with systolic blood pressure ranging from 140 to 184 mmHg. Other vital signs were within a normal range including body temperature. Her body mass index was 30. Laboratory tests were remarkable for hemoglobin of 8.7 g/dl, erythrocyte sedimentation rate (ESR) of 33 mm/hour, and urinalysis of +1 proteinuria. She was diagnosed with preeclampsia. The mild ESR elevation was attributed to pregnancy. Betamethasone (a total of two 12-mg intramuscular injections one day apart) was given to enhance the fetal lung maturity for anticipated preterm delivery. She developed acute bilateral knee pain within hours of the second dose of intramuscular betamethasone. The pain was sharp, moderate to severe in intensity, and associated with a tingling sensation over bilateral anterior thighs and knees. The pain was aggravated by movement and walking. She was initially managed with close monitoring and Tylenol as needed for pain. There were no reported similar symptoms prior to betamethasone administration and no prior imaging.

**Figure 1:**
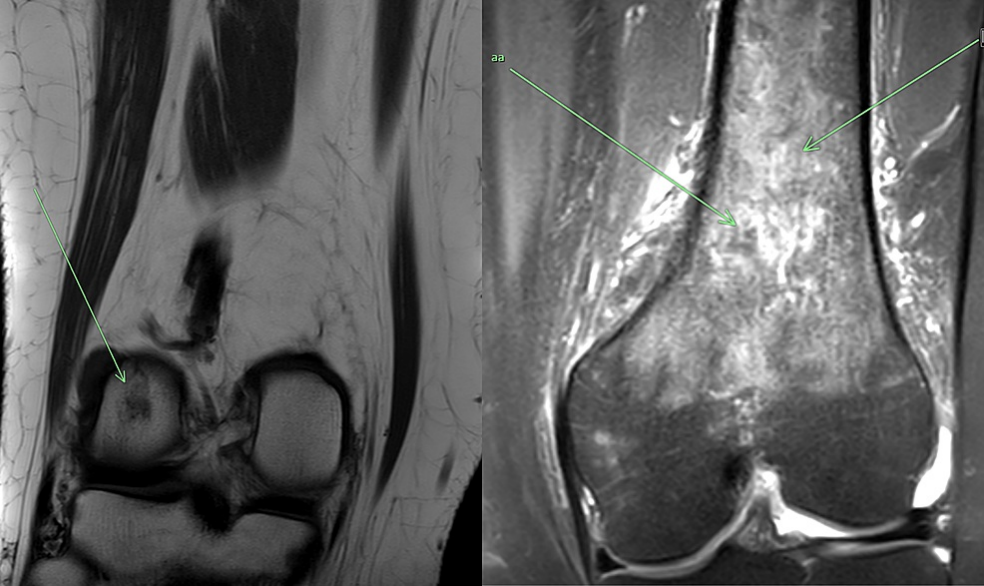
Image on the right with green arrows: MRI of the knee joint showing circumferential periosteal edema involving bilateral distal femoral meta diaphysis, which is suggestive of medullary infarcts. Image on the left with a green arrow: MRI of the right knee showing an area of bone marrow edema-like signal that represents avascular necrosis at the lateral femoral condyle.

Two days later, she underwent uncomplicated vaginal delivery of two baby girls. She continued to have bilateral knee pain, at rest, more so with movement which made her unable to ambulate. Her vital signs were within normal range after delivery, and her laboratory tests were unchanged. On physical examination of bilateral lower extremities, there was tenderness over bilateral distal thighs and knees. There was no erythema, joint swelling, or warmth. The range of movement of bilateral hip and knee joints was intact. The sensation was intact over bilateral lower extremities. Orthopedic surgery and rheumatology teams were involved in the patient care. X-rays of bilateral distal femurs and knees were unremarkable. Magnetic resonance imaging (MRI) of bilateral lower extremities revealed circumferential periosteal edema involving bilateral distal femoral meta diaphysis, which is suggestive of medullary infarcts and showed a tiny focal subchondral marrow signal abnormality measuring up to 6 mm at the posterior aspect of the right lateral femoral condyle, which reflects a tiny focus of avascular necrosis without associated articular surface collapse (Figure 1).

She has no known medical problem apart from combined sickle cell trait and beta-thalassemia trait with no reported history of pain crisis or blood transfusion. She has never received steroid therapy in the past and has no tobacco or alcohol use. She reported a family history of hypertension and coronary artery disease with no family history of autoimmune diseases. Prior HIV test was negative.

She was diagnosed with osteonecrosis of bilateral femurs secondary to betamethasone therapy. She was treated conservatively with analgesics and instructed to avoid strenuous activities and prolonged weight-bearing. The patient was advised to follow up with orthopedic surgery after discharge. Two months later, on a follow-up appointment with her gynecologist, she was still complaining of bilateral knee pain with ambulation, and she failed to follow up with orthopedic surgery or gynecology thereafter.

## Discussion

We present a case of osteonecrosis of the bilateral distal femurs and right lateral femoral condyle that started shortly following the second of two doses of intramuscular betamethasone injections in a pregnant woman with combined sickle cell trait and beta-thalassemia trait. The patient did not have signs of SLE, and she had no history of prior trauma, knee surgery, HIV, bisphosphonates, or other antiresorptive medications use, and no alcohol, or tobacco use. She also had no signs of concurrent infection.

Avascular necrosis of the femoral head of the hip during pregnancy has been reported in less than 100 cases [12], and no cases of osteonecrosis affecting femoral meta diaphysis and/or condyle have been reported in pregnancy to our knowledge. Although osteonecrosis is a well-known complication of sickle cell disease (prevalence of which increases with the presence of coexisting alpha-thalassemia trait) [13], it has been reported in only a few cases with sickle cell trait and sickle-beta-thalassemia trait, with osteonecrosis primarily affecting the femoral head in these cases [14,15].

Finally, while spontaneous/primary osteonecrosis of the bones around the knee joint has been documented, we were unable to locate any cases of primary osteonecrosis of the bilateral distal femurs [16]. After ruling out the alternative possibilities, osteonecrosis was attributed to steroid medication in our case due to the chronological association. No prior imaging was done to her lower extremities to compare. Sickle cell trait, beta-thalassemia trait, and weight gain during pregnancy could have been the underlying intrinsic risk factors for osteonecrosis in our patient. Lesser-known intrinsic risk factors for osteonecrosis could have been the twin pregnancy and/or preterm delivery in our patient.

Early signs of osteonecrosis were detected by MRI after negative x-rays in our case. Early-stage osteonecrosis can be diagnosed using MRI, which is the gold standard test. Conventional radiographs would detect later stages of bone destruction. A variety of management strategies have been developed depending on the extent of osteonecrosis. The conservative approach includes analgesics, minimizing weight-bearing, and avoidance of vigorous physical exercise. Some of the surgical options include core decompression with and without bone graft and joint arthroplasty. Unfortunately, we were unable to monitor our patient's condition or determine the outcome because she did not return for follow-up.

## Conclusions

Osteonecrosis has been rarely reported in association with pregnancy and sickle beta-thalassemia traits. Prior reported cases of osteonecrosis during pregnancy had primarily involved the femoral head, and according to our literature review, we could not identify reported cases of femoral meta diaphyseal osteonecrosis during pregnancy similar to what we are presenting in this case. Steroid therapy is a known risk factor for osteonecrosis, more so with long-term use and high cumulative dose.

In this study, we reported a rare presentation of osteonecrosis after antenatal betamethasone injection, which involved unreported location in pregnancy. This indicates the need to have a high level of suspicion when evaluating pregnant patients with unexplained musculoskeletal pain, especially after corticosteroid therapy even if the symptoms start shortly after one or two doses, keeping in mind that osteonecrosis is not restricted to the femoral head and can affect other sites such as the distal femoral meta diaphysis and femoral condyle. Sickle and beta-thalassemia traits could have been the triggering factors for this unfavorable effect of corticosteroid treatment. To further understand the risk factors, correlations, and prognosis of steroid-induced osteonecrosis in pregnancy, more research is needed. Prospective studies following patients receiving steroid therapy (including brief courses) can help to detect osteonecrosis complications and to check for associations and clinical courses.
